# A constitutive expression system for glycosyl hydrolase family 7 cellobiohydrolases in *Hypocrea jecorina*

**DOI:** 10.1186/s13068-015-0230-2

**Published:** 2015-03-18

**Authors:** Jeffrey G Linger, Larry E Taylor, John O Baker, Todd Vander Wall, Sarah E Hobdey, Kara Podkaminer, Michael E Himmel, Stephen R Decker

**Affiliations:** Biosciences Center, National Renewable Energy Laboratory, 16253 Denver West Parkway, Golden, CO 80401 USA; National Bioenergy Center, National Renewable Energy Laboratory, 16253 Denver West Parkway, Golden, CO 80401 USA

**Keywords:** *Hypocrea jecorina*, *Trichoderma reesei*, Cellobiohydrolase, Cellulase expression, Fungal molecular biology, Biomass hydrolysis

## Abstract

**Background:**

One of the primary industrial-scale cellulase producers is the ascomycete fungus, *Hypocrea jecorina*, which produces and secretes large quantities of diverse cellulolytic enzymes. Perhaps the single most important biomass degrading enzyme is cellobiohydrolase I (*cbh1*or Cel7A) due to its enzymatic proficiency in cellulose depolymerization. However, production of Cel7A with native-like properties from heterologous expression systems has proven difficult. In this study, we develop a protein expression system in *H. jecorina* (*Trichoderma reesei*) useful for production and secretion of heterologous cellobiohydrolases from glycosyl hydrolase family 7. Building upon previous work in heterologous protein expression in filamentous fungi, we have integrated a native constitutive enolase promoter with the native *cbh1* signal sequence.

**Results:**

The constitutive *eno* promoter driving the expression of Cel7A allows growth on glucose and results in repression of the native cellulase system, severely reducing background endo- and other cellulase activity and greatly simplifying purification of the recombinant protein. Coupling this system to a Δ*cbh1* strain of *H. jecorina* ensures that only the recombinant Cel7A protein is produced. Two distinct transformant colony morphologies were observed and correlated with high and null protein production. Production levels in ‘fast’ transformants are roughly equivalent to those in the native QM6a strain of *H. jecorina*, typically in the range of 10 to 30 mg/L when grown in continuous stirred-tank fermenters. ‘Slow’ transformants showed no evidence of Cel7A production. Specific activity of the purified recombinant Cel7A protein is equivalent to that of native protein when assayed on pretreated corn stover, as is the thermal stability and glycosylation level. Purified Cel7A produced from growth on glucose demonstrated remarkably consistent specific activity. Purified Cel7A from the same strain grown on lactose demonstrated significantly higher variability in activity.

**Conclusions:**

The elimination of background cellulase induction provides much more consistent measured specific activity compared to a traditional *cbh1* promoter system induced with lactose. This expression system provides a powerful tool for the expression and comparison of mutant and/or phylogenetically diverse cellobiohydrolases in the industrially relevant cellulase production host *H. jecorina*.

## Background

Enzymatic deconstruction of biomass to liberate monomeric sugars for the biological production of fuels and chemicals has been a research direction of global importance over the last several decades. One of the primary industrial-scale cellulase producers is the ascomycete fungus, *Hypocrea jecorina*, which produces and secretes large quantities of diverse cellulolytic enzymes. *H. jecorina* is not as genetically malleable as many other microorganisms, making it a challenging organism to use as a tool for the manipulation and expression of heterologous enzymes. However, recent work has expanded the tools available for genetically manipulating *H. jecorina*, including enhanced homology-based gene targeting via disruption of the non-homologous end joining (NHEJ) pathway [1,2], reusable genetic markers [1,3], strong constitutive promoters [4], and sexual crossings [5,6]. Yet, even with these advances, the genetic system of *H. jecorina* presents significant technical challenges when compared to other model microbial organisms.

A significant body of research has been focused on expression of *H. jecorina* enzymes in heterologous hosts. One enzyme that has received particular focus is cellobiohydrolase I (the gene is referred to as ‘*cbh1*’ and the protein as ‘Cel7A’), due to its enzymatic proficiency in cellulose depolymerization. However, production of Cel7A with native-like properties from heterologous expression systems has proven difficult. For example, Cel7A expression in *Pichia pastoris* yielded hyperglycosylated and misfolded protein with reduced activity [7,8], expression in *Ashbya gossypii* yielded catalytically inactive enzyme [9], and expression in *Aspergillus niger* var. *awamori* produced over-glycosylated isoforms with reduced activities and altered thermal stability [10]. Numerous expression studies of *H. jecorina* Cel7A in *Saccharomyces cerevisiae* also show hyperglycosylation, low-level expression, and/or low-level secretion, although some other fungal cellobiohydrolases appear more amenable to yeast expression [11,12]. Dana *et al.* [13] have recently shown that this result is at least in part due to the failure of *S. cerevisiae* to correctly process the *N*-terminal glutamine of Cel7A. Whereas there have certainly been advances in the heterologous expression and secretion of cellobiohydrolases in yeast [14-19], the overall trend is clear - there remains a significant challenge in effectively expressing Cel7A enzymes in organisms other than the native species.

As Cel7A is the major enzymatic activity in the *H. jecorina* cellulase system, the wide variety of issues with heterologous expression of Cel7A is a significant concern for cellulase improvement. Without a simple, robust, and productive heterologous expression system capable of producing Cel7A with native characteristics, improvement of Cel7A for inclusion in new industrial cellulase formulations becomes very difficult. Because *H. jecorina* is a major commercial cellulase production host and because Cel7A produced by other heterologous hosts is not necessarily equivalent to *H. jecorina*-produced Cel7A, evaluating novel or engineered enzymes produced by *H. jecorina* itself promises to be a very valuable tool. However, using *H. jecorina* as an expression host for recombinant Cel7A presents additional problems. With the objective of engineering a single cellulase, it is imperative that the enzyme of choice be produced in an enzymatically ‘clean’ background. Many cellulase expression studies in *H. jecorina* use the powerful *cbh1* promoter, which is induced by the presence of many substrates, including lactose, cellulose, and sophorose (reviewed in [20]). However, the induction of Cel7A expression also results in the induction of the entire cellulase system, making the *cbh1* promoter less than ideal for expressing single enzymes. Moreover, in order to achieve very high titers of cellulases, research is frequently conducted on highly mutated strains, such as RUT-C30, which are extremely proficient enzyme producers. These de-repressed strains constitutively express large suites of enzymes, even when grown on glucose, making the detailed study of single enzymes difficult. Growing the wild-type strain, QM6a, on glucose results in complete repression of the cellulase system. The use of QM6a as an expression host has the distinct advantage of allowing high expression of the target heterologous protein while repressing expression of other cellulases.

Obviously, an *H. jecorina* strain in which the endogenous cellulases are deleted would be ideal for production and characterization of heterologous cellulases. However, given the slow nature of sequential gene deletion in *H. jecorina* and the sheer number of potentially ‘contaminating’ cellulases produced by this host, we instead worked to generate an expression system that would utilize catabolite repression of endogenous cellulases while providing robust expression of our single target cellulase, in this case, Cel7A. As glucose is a natural global repressor of the cellulolytic machinery in *H. jecorina* [21], with repression mediated through the Cre1 repressor protein [22], the use of promoters with strong activity in glucose-containing media provides a valuable tool for the simultaneous expression of singular enzymes with the global repression of endogenous cellulases. For example, *Tef1* was identified as a strong promoter in glucose-containing media [23] and was successfully used to drive expression of both Cel7A and EGI (endoglucanase I) [24]. Recently, it was shown that the promoters from two glycolytic pathway enzymes, namely, enolase and pyruvate decarboxylase, were constitutively active in glucose-containing media and were capable of expressing high levels of homologous xylanases in *H. jecorina* [4]. While the *pdc* promoter was reported to be slightly better than *eno* (83 *vs*. 82% of total protein), the level of precision of the densitometry used to measure relative protein levels is low enough that the two promoters may be considered equivalent in performance [4]. We chose to use *eno* initially and since it functions remarkably well for Cel7A expression, we have not pursued using the *pdc* promoter.

For the immediate purpose of expressing native and engineered Cel7A homologously, and with the long-term goal of developing an expression system generally capable of expressing and secreting important classes of single proteins, we utilized a QM6a strain deleted for the native *cbh1* gene as the host and used an integrating expression cassette driving the expression of Cel7A from the *eno* promoter. Using this system, we are capable of producing native Cel7A with very little, if any, background from native cellulases. This report describes, to the best of our knowledge, the first successful use of a glucose-active promoter to drive Cel7A expression in a *cbh1* deletion strain. Accordingly, this work represents a technical foundation for moving towards our ultimate goal of generating a robust cellulase expression and secretion host for detailed expression studies on various classes of glycolytic enzymes in *Trichoderma reesei*. While Li *et al*. expressed a xylanase in a similar system, they did not purify the enzyme to test its intrinsic kinetic properties or compared it to xylanases expressed in their native context [4].

## Results and discussion

**Figure 1:**
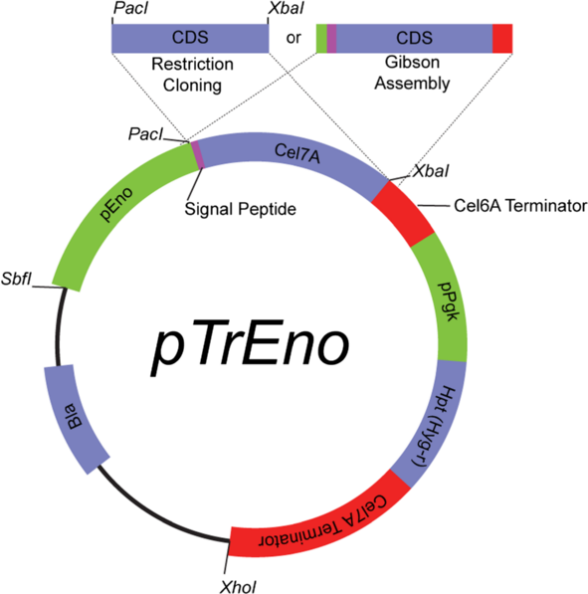
**Schematic and features of the pTrEno expression plasmid.**

We set out to create a heterologous host capable of high levels of cellulase expression in the absence of contaminating endogenous cellulases. To achieve this goal, we replaced the *cbh1*-promoter sequence in our vector pTR50 [25] with the *eno* (enolase) promoter to generate a vector, called ‘pTrEno’ (Figure 1). Enolase is a glycolytic enzyme whose transcriptional level is constitutive in glucose-containing medium [4], a situation which simultaneously serves to repress endogenous cellulases. Furthermore, to avoid even the smallest amount of contaminating native Cel7A, we used strain AST1116, a QM6a derivative strain, deleted for the native *cbh1* gene, as our host strain. To summarize, this newly generated strain, JLT102A, has the native *cbh1* gene deleted and a chromosomally integrated *pEno-cbh1* cassette liberated from pTrEno.

The pTrEno vector was designed such that the *eno* promoter can be readily substituted and the coding sequence can be easily interchanged using either traditional restriction cloning or recombination-based cloning techniques, including Gibson Assembly [26]. As the *cbh1* 5′ homology region was deleted during the construction of pTrEno, the expression cassette liberated by restriction digest with *SbfI* and *XhoI* does not target via homology and instead serves as a random integration cassette. While specific chromosomal integration sites are more difficult to identify, non-homologous integration allows multiple cassettes to be incorporated, a common phenomenon in *H. jecorina* [27,28]. Furthermore, random insertion can provide the mechanism for integrating into chromosomal transcriptional ‘hot-spots,’ such as euchromatic regions potentially enabling heightened expression, while avoiding transcriptionally repressed heterochromatic regions [29]. Chromatin heterogeneity is found in virtually all eukaryotes from *S. cerevisae* to humans and can have dramatic effects on gene expression. Such site-specific integration effects on gene expression will likely be observed with both the cellulase genes and the antibiotic resistance gene (*hph* in this case) contained on the expression cassette. Accordingly, expression can be variable from clone to clone, and random integration can lead to strains with heightened expression. We have thus designed a versatile plasmid for high-level expression of homologous or heterologous enzymes in *H. jecorina* in the absence of endogenous cellulase expression*.*

**Figure 2:**
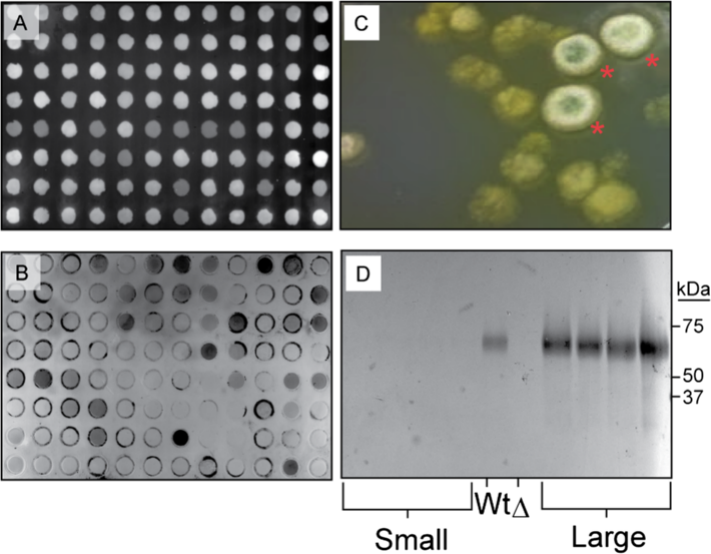
**Rapid screening of the secretomes of potential transformants.** Colonies from transformed plates were allowed to grow in liquid medium for 3 days prior to being screened by Western blot. **(A)** PVDF membrane illuminated with UV light to indicate successful transfer of broth and proteins to membrane. **(B)** Anti-Cel7A Western blot on the membrane shown in (A), showing numerous immunoreactive transformants. **(C)** We identified both ‘small’ and ‘large’ colonies after allowing transformed plates to incubate for beyond 3 days. **(D)** SDS-PAGE coupled Western blot highlights our observations that ‘large’ colonies are overwhelmingly more likely to be true Cel7A transformants expressing protein.

After designing the plasmid, we next wanted to determine the transformation and expression efficiency of the pTrEno expression construct. *H. jecorina* strain AST1116 was transformed via electroporation and plated onto potato-dextrose agar plates with hygromycin for selection and the non-ionic, non-denaturing detergent, IGEPAL CA-630, as a colony size restrictor. Two distinct colony morphologies are observed during transformation: ‘fast growers’ and ‘slow growers’ (Figure 2C), where only fast growers appear to have the potential to be enzyme-expressing transformants. Yet, even within the fast-growing subset, screening the extracellular glucose-containing growth medium of fast-growing transformants by dot-blot protein immunoblot showed varied expression between transformants (Figure 2A,B). As suggested above, this result could be due to multiple integrations of the expression cassette or to chromosomal position effects. Sodium dodecyl sulfate polyacrylamide gel electrophoresis (SDS-PAGE) coupled with Western blot analysis confirms the results of the initial dot-blot screen, where four-of-four fast growers show a protein band consistent with Cel7A as compared with an immune-reactive band from wild-type QM6a grown in lactose-containing medium for cellulase induction. Slow growers produce no immuno-reactive proteins (Figure 2D).

**Figure 3:**
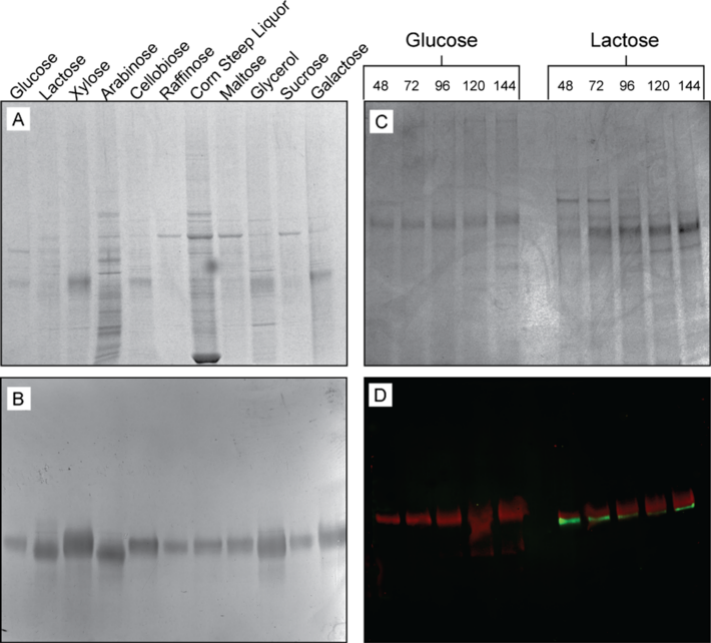
***eno-*** **driven Cel7A is constitutively expressed using numerous sole carbon sources and Cel6A is not expressed in glucose medium. (A-B)** The same stock of JLT102A was used to inoculate media with varied carbon sources. Following 3-day growth, the secretome was analyzed via SDS-PAGE coupled Western blots. **(A)** Amido black staining highlights the total extracellular protein in each described media. **(B)** Anti-Cel7A Western blots show Cel7A expression in each described medium. **(C-D)** JLT102A was grown in MAG or MAL, and a time course of medium was taken for Western blot analyis using both Cel7A and Cel6A coupled with differently colored fluorescent secondary antibodies. **(C)** Amido black-stained PVDF membrane showing total protein as a loading control. **(D)** Multiplex Western blot using anti-Cel7A (red) and anti-Cel6A (green) shows constitutive *eno*-Cel7A expression and glucose repression of endogenous Cel6A.

Given the constitutive nature of the *eno* promoter, we next wanted to determine *eno****-***driven Cel7A expression in media containing various carbon sources. The changes in composition of total protein with changes in carbon source are shown in Figure 3A. Using Western blot, it was found that Cel7A is expressed in all media tested (Figure 3B). Interestingly, the relative amounts of Cel7 vary by carbon source, in that it appears the use of xylose or glycerol specifically produces higher levels of Cel7A in a cleaner background. However, this preliminary observation should be confirmed in a more quantitative manner. Additionally, the apparent molecular weight distribution of Cel7A is slightly shifted between the various carbon sources, perhaps a consequence of differential glycosylation characteristics of the protein [30]. Further investigation of the fundamental differences in Cel7A purified from strains grown on various carbon-containing media will be of particular interest for optimizing this process.

Of primary importance to the design of this expression system was the notion that the strain should produce very little to no endogenous cellulases during the production of the *eno*-driven Cel7A. To examine this concern specifically, we performed a time-course growth of JLT102A coupled with multiplex Western blot using antibodies directed towards both Cel7A and Cel6A (*cbhII*) to analyze protein contents of extracellular growth medium containing either glucose or lactose (Figure 3C,D). Cel6A was specifically examined because it is second only to Cel7A in abundance in the *H. jecorina* secretome [31]. As expected, Cel7A was expressed in either glucose- or lactose-containing medium, whereas Cel6A could only be detected in lactose-containing medium. This lack of endogenous cellulases makes the purification of Cel7A much simpler and reduces the risks of cross-cellulase contamination during the measurement of activities. This outcome is of the utmost importance, as we move towards exploring and rapidly assessing phylogenetically diverse and mutant enzymes expressed in this *H. jecorina* system.

**Figure 4:**
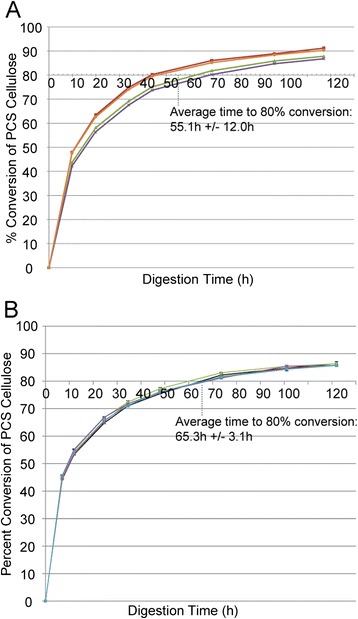
***eno-*** **expressed Cel7A provides consistent reproducibility in enzyme activity using pretreated corn stover as a substrate. (A)** Four independent preparations of Cel7A from QM6a show very high levels of variability from batch to batch making enzyme activity assessments difficult. **(B)** Five independent preparations of *eno*-expressed Cel7A show remarkable consistency in enzyme activity assays. For both curves, error bars from triplicate assay digestions are included but are very difficult to see owing to the highly reproducible nature of these digestions.

To validate that Cel7A enzyme produced from the *eno* promoter was functionally active, we concentrated the secreted enzyme from the growth medium and purified the enzyme using multiple fast protein liquid chromatography protocols. Enzymatic activity assays were carried out using dilute acid pretreated corn stover as the substrate. As can be seen in Figure 4, the performance of the *eno*-driven Cel7A (Figure 4B) is consistent with that of Cel7A purified from the wild-type QM6a secretome (Figure 4A).

Previous work in our lab evaluating wild-type rCel7A from a *cbh1*-delete, RUT-C30-based expression system [25] as well as native QM6a and RUT-C30 Cel7A protein from lactose fermentations, resulted in inconsistent specific activities on dilute acid-pretreated corn stover (Figure 4A) when assayed in a ternary enzyme system [32]. These results suggest either an inconsistency in protein processing, that is, glycosylation or trimming, or that purification from the high-cellulase background was itself variable, with low-levels of background endocellulase activity leading to variable observed activity. As the main goal of our work is to measure changes in Cel7A activity as a result of genetic manipulations and to screen new Cel7 exocellulases for enhanced properties, inconsistent measured activity from independent growth and purification steps was of great concern. To demonstrate that use of our new *eno*-driven expression system avoids this problem, we purified Cel7A from five independent *eno*-driven Cel7A fermentations, each grown under exacting conditions to minimize the impact of growth/stress parameters on enzyme activity. High stringency hydrolysis assays of these five purified proteins on pretreated corn stover clearly demonstrate nearly identical activities (Figure 4B). While the underlying mechanism(s) of activity inconsistency for the RUT-C30-strain-expressed Cel7A is still not entirely clear, the pTrEno system clearly demonstrates a much more consistent and stable system for evaluating differences in cellobiohydrolase activity.

**Figure 5:**
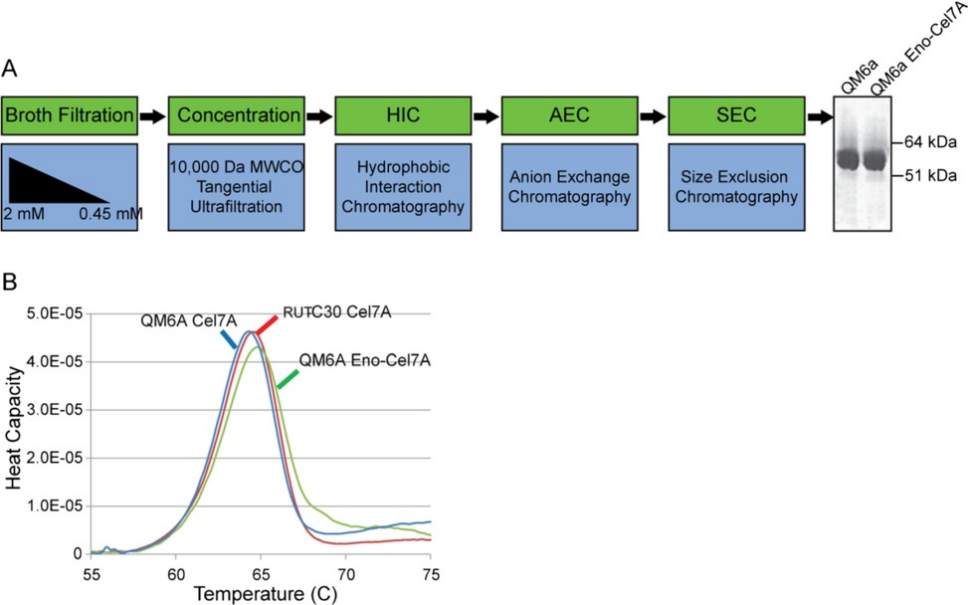
**Cel7A purification schematic and thermal stability comparisons. (A)** Purification schematic. **(B)** Differential scanning calorimetry (DSC) to determine the thermal stability of *eno*-driven Cel7A compared to wildtype Cel7A derived from QM6a and RUT-C30.

Biophysical characterization of rCel7A was carried out for comparison with the native enzyme. Thermal stability was evaluated by differential scanning microcalorimetry (DSC). The eno-expressed protein showed no significant difference in thermal stability compared with the wild-type (Figure 5B), unlike Cel7A expressed from *Saccharomyces* or *Aspergillus*, which have shown significant differences compared to the wild-type Cel7A [33,34]. Similarly, the molecular weights of these enzymes as determined by SDS-PAGE (Figure 5A) show very similar masses for QM6a- and *eno*-expressed Cel7A, whereas molecular weights of *Aspergillus*- and *Saccharomyces*-expressed Cel7A are significantly higher, presumably due to increases in glycosylation.

Accuracy and consistency are important attributes of any expression system meant to compare enzyme activities. However, achieving this capability has proven very challenging in typical *cbh1*-promoter-driven systems using lactose as the inducer. Specifically, in our hands, growth and enzyme expression have proven quite variable using this system from trial to trial. For example, Figure 4A shows drastically different enzyme activities from independently expressed and purified enzymes from lactose-containing medium, even though they are identical in amino acid sequence. In contrast, when we performed five independent expressions and purifications of Cel7A derived from the *eno*-driven system, we see remarkable consistency in enzyme activity (Figure 4B). We suspect that much of the inconsistency with the lactose-induced wild-type Cel7A activity profiles arises from inconsistent purification despite the use of a rigorous purification scheme (Figure 5A). Miniscule amounts of other endogenous cellulases can drastically swing these PCS digestion curves making it very difficult to compare enzyme activities using this system. However, using the *eno*-driven system, much of this inconsistency falls away. For example, the average time it takes to achieve 80% digestion of PCS using four independently purified Cel7A preps from QM6a in lactose-containing medium is 55.1 h with a standard deviation of 12.0 h. In contrast, five independently generated preparations of *eno*-driven Cel7A in glucose-containing medium take an average of 65.3 h. However, the standard deviation of this latter set is only 3.1 h. Given the variable and unpredictable nature of our Cel7A preparations from QM6a in lactose-containing medium, an exact comparison of the *eno*-driven Cel7A with the wild type form is exceedingly difficult to perform with any confidence. However, for future assessments of enzyme activities, the consistency provided by the *eno*-driven system will be of paramount importance in studies aimed at comparison of heterologous or mutant enzymes.

The work presented here springboards off of the work of Li *et al*. [4] and provides more functional characterization of the enzymes expressed. To summarize, *Li et al*. quite clearly showed that the *eno* promoter (among others) was capable of expressing a single xylanase to high levels and represents the initial identification and application of a powerful tool for *T. reesei* molecular biology-based pursuits. However, this enzyme was not purified, and there were no biophysical or enzymatic activity comparisons to the native enzymes. This data is critical to understanding the effect of altering the gene expression environment (that is, promoter, carbon source) on the activity and biophysical state of the expressed enzyme. As such, the work presented here adds much-needed validation of the use of glycolytic promoters for the expression of cellulolytic enzymes in *H. jecorina* and provides a valuable tool to the field of fungal enzyme expression.

## Conclusions

The portfolio of biomass-derived fuels and chemicals continues to expand, and accordingly, the ability to efficiently depolymerize cellulose remains a critical industrial challenge. Consequently, the need for identifying superior enzymes continues to be a priority, and perhaps no enzyme class is as valuable to this end goal as are the cellobiohydrolases. However, there are very few heterologous expression systems that produce enzymes functionally equivalent to wild-type enzymes and are free from contaminating endogenous cellulases. The system we report here, using a glycolytic (*eno*) promoter-driven processing construct using the *cbh1* signal sequence in a *cbh1* deletion strain, provides a platform for detailed analysis of single heterologous cellulases produced in a native, industrially relevant host. Standard activity assays of heterologously expressed Cel7A show that these enzymes are comparable to wild-type Cel7A on pretreated corn stover and have similar thermal stability and glycosylation. More importantly, the eno-expression system permits heterologous Cel7A expression while repressing native cellulase production, making protein purification easier and, critically, eliminating variability in measured activity possibly caused by synergy with trace amounts of endocellulases. Future work will focus on increasing expression protein levels, exploiting the expression of targeted and random mutations, and exploring the field of incorporating phylogenetically diverse enzymes into the *H. jecorina* secretome.

## Methods

### Media and growth conditions

Growth medium for Cel7A expression was a modified version of Mandels and Andreotti (MA) medium [35]. To make 1.0 L of MA, add 20.0 mL 50× MA salts, 5.0 g tryptone up to 737 mL with H_2_O. Autoclave and then add 2.7 mL of separately sterilized 1.0 M CaCl_2_ to minimize precipitation. Add 10 mL of filter sterilized micronutrient solution. Add 250 mL sterile 20% glucose or lactose to make 1.0 L of Mandels Andreotti minimal medium with 5% glucose (MAG) or Mandels Andreotti minimal medium with 5% lactose (MAL), respectively. Add hygromycin to a final concentration of 100 μg/mL as needed.

To prepare 1.0 L of 50× MA salts: combine 100.0 g KH_2_PO_4_, 70.0 g (NH_4_)_2_SO_4_, 15.0 g urea, and 15.0 g MgSO_4_.7H_2_O, titrate to pH 5.5 with KOH. To prepare 1.0 L of micronutrient solution, add 500 mg FeSO_4_.7H_2_O, 160 mg MnSO_4_.H_2_O, 140 mg ZnSO_4_, and 200 mg CoCl_2_. Dissolve each component completely in order listed and then filter sterilize.

Growth medium for transformation outgrowth was complete medium lactose (CML), which consisted of 5.0 g/L yeast extract, 5.0 g/L tryptone, and 10.0 g/L lactose in a volume of 950 mL. The pH was adjusted to pH 7.5 with KOH and autoclaved. Following cooling, 50 mL of Clutterbuck’s salt solution (per L: 120.0 g NaNO_3_, 10.4 g KCl, 10.4 g MgSO_4_, 30.4 g KH_2_PO_4_) was added. For spore production, potato dextrose (PD) plates were used and made according to the manufacturer’s (Sigma Aldrich, St. Louis, MO, USA) recommendations. Hygromycin was added to the medium (after autoclaving) at a concentration of 100 μg/mL to make ‘PDH’ plates ‘as required’ following transformation, and IGEPAL CA-630 (similar to TritonX-100) was added at 0.1% as a colony restrictor to make ‘PDHX’ plates.

### pTrEno construction

Vector pTR50 was PCR-amplified excluding the *cbh1* promoter and upstream homology region using primers (fwd: ATGTATCGGAAGTTGGCCGTC, rev: TCTCGACGCATTCGCGAA). The *eno* promoter was amplified directly from gDNA extracted from QM6a using primers (fwd: TTCGCGAATGCGTCGAGA*CCTGCAGG*-tgattccgtcctggattgc, rev: GACGGCCAACTTCCGATACAT*TTAATTAA*-tttgaagctatttcaggtggctgg).

These primers have 5′ ‘tails’ (capitalized) that are homologous to the ends of the PCR-linearized pTR50 described above and have the *SbfI* and *PacI* restriction sites incorporated, respectively (italicized). *In vitro* recombination was achieved using Gibson Assembly (New England Biolabs, Ipswich, MA, USA) according to the manufacturer’s protocol.

### PAGE

Culture broths were clarified via centrifugation and transferred to microcentrifuge tubes. Broths were diluted 3:1 in 4× LDS sample buffer (Life Technologies Corp., Carlsbad, CA, USA) with 50 μL/mL β-mercaptoethanol as a reducing agent. Samples were incubated at 95°C for 5 min prior to loading onto NuPAGE SDS gels with MOPS buffer, electrophoresed at 200 V constant for approximately 50 min and then transferred to a polyvinylidene difluoride (PVDF) membrane for Western blotting using the iBlot transfer system (Life Technologies Corp., Carlsbad, CA, USA).

### Dot-blotting

Ninety-six-well glass fiber filter plates (Millipore Corp., Billerica, MA, USA) were used to clarify growth medium of *H. jecorina* cultures. Three hundred microliters of broth was centrifuged for 1 min (2,000 × *g*) through the filter plate into 96-well receiver plates. PVDF membranes were cut to fit the dot blot apparatus, soaked in methanol for approximately 1 min, washed for 1 min in distilled water, and then overlaid on wetted Whatman filter paper cut to the same size and assembled on the dot blot apparatus. Typically, 100 μL of broth was loaded into each well, and a vacuum was applied until each well had the entirety of the broth pulled through. Blots were allowed to air dry and then were visualized and imaged under UV light (Fluorchem Q, Protein Simple, San Jose, CA, USA) to assure membrane transfer. Total protein detection is not achievable using this method due to the background fluorescence of media components. Blots were then reactivated in methanol and analyzed by Western blot.

### Western blots

For single antibody Western blots (Figures 2B,D and 3B), immuno-detection of Cel7A was achieved using the SNAP i.d. Protein Detection System (Millipore Corp.). The PVDF membrane was blocked using SuperBlock PBS (Thermo Fisher Scientific Inc., Rockford, IL, USA) for 20 min. Rabbit anti-Cel7A polyclonal IgG was used as the primary antibody (1:20,000 dilution of crude serum), with alkaline phosphatase-conjugated goat anti-rabbit IgG (Thermo Fisher Scientific Inc.) as secondary. The alkaline phosphatase localization was visualized using BCIP/NBT (Life Technologies Corp., Carlsbad, CA, USA).

For the multiplex Western blot shown in Figure 3D, all solutions were sterile filtered to minimize background fluorescence. SDS-PAGE gels were transferred via standard wet tank transfer to PVDF membranes. All post-transfer solutions were from Protein Simple. Membranes were blocked in blocking solution for 1 h at room temperature and then washed four times for 2 min each in wash buffer. Polyclonal rabbit anti-Cel7A and monoclonal mouse anti-Cel6A antibodies were diluted 1:5,000 in blocking buffer, and the blot was incubated for 1 h. The blots were washed four times for 5 min in wash buffer. Goat anti-rabbit (red) and goat anti-mouse (green) Alexa Fluor conjugated antibodies were diluted 1:1000 in blocking buffer, added to the PVDF membrane and incubated at room temperature and covered to protect from ambient light with orbital shaking for 1 h. Membranes were washed three times for 10 s, followed by four washes for 5 min in wash buffer and finally washed two times for 5 min in final wash buffer. The blots were allowed to dry and then visualized using a FluorchemQ imaging system (Protein Simple). The total blot contrast was digitally adjusted evenly across the image planes to ensure ease of visualization of the bands.

### Growth conditions

Small-scale growths were conducted in shake flasks at 30°C at 225 RPM in either MAG or MAL medium for 2 to 3 days. For Cel7A purification, *H. jecorina* spore stocks were streaked on potato dextrose agar plates and allowed to grow 2 to 3 days until a well ‘lawned’ plate of spores was achieved. Using the wide end of a sterile 1.0-mL pipette tip, an approximately 0.5-cm plug was extracted from the plate and deposited into 1.0 L of MAG medium in a 2.8-L shake flask. The culture was grown at 28°C with at 225 RPM for 24 h, after which the entire 1.0 L was transferred to 7.0 L of the same medium in a bioreactor. The bioreactors were 10-L working volume vessels manufactured by New Brunswick (Eppendorf Inc., Enfield, CT, USA) and controlled via New Brunswick’s BioFlo3000 system. The total of 8.0 L was grown with mixing at 200 RPM with a combined Rushton (upper) and marine downflow (lower) style impellers (Rushton and Company, Gainesville, GA, USA), purged with 1.0 vol*vol^-1^*min^-1^ of filtered air, kept at a strict 28°C, and pH controlled at 4.8 for 48 h. pH control was accomplished using 2.0 M HCl and KOH.

### Transformation procedure

Transformations were achieved by way of spore electroporation, as modified from [6]. Spores from a frozen stock were spread evenly onto PD agar plates and allowed to grow at 30°C in the light (to enhance sporulation) for 2 to 3 days. The spores were harvested by gently spreading 2.0 to 3.0 mL ice-cold sterile distilled water on the plates to suspend the spores. The spores were moved to microcentrifuge tubes and centrifuged for 5 min at 2,000 × *g* at 4°C. The spores harvested from up to five plates were pooled and suspended in 1.0 mL of ice-cold 10% glycerol. The spores were either used immediately for transformation or frozen at −80°C for future use. For transformation, 5 μg of pTrEno was digested with *SbfI* and *XhoI* and gel purified to isolate the Cel7A expression cassette. Purified DNA in 10 μL of water was mixed with 100 μL of spore suspension and placed in an ice-chilled 0.1-cm gap electrocuvette. The spores were electroporated using a BioRad Gene Pulser (Bio-Rad, Hercules, CA, USA) and the following conditions (1.8 kV, 25 μF, 800 Ω). Immediately following pulse, 1.0 mL of CML was added to the transformation. This cell suspension was then transferred to six-well tissue culture plates and incubated statically on a benchtop overnight (approximately 18 h). Microscopic visualization of the spores following this incubation shows spores just beginning to elongate and enter an active cellular growth stage. At this point, the cells were suspended by repeatedly pipetting up and down, and then 100 μL of cell suspension was plated onto potato dextrose with hygromycin and Triton X-100 (PDHX) plates. Transformants were allowed to grow for 2 to 3 days at 30°C in lighted incubators to enhance sporulation. Fast-growing colonies were selectively picked by hand for Cel7A expression testing. The selected colonies grew more rapidly and began to sporulate and turn green at a much faster rate compared to the slow-growing background colonies (Figure 2).

### Initial screening of transformants and generation of clonal stocks

To screen initial single-colony transformants, ‘plugs’ were cored from agar plates using sterile disposable Pasteur pipettes and transferred to 2.0 mL of MAG medium supplemented with hygromycin (100 μg/mL) in sterile 24-well tissue culture plates. Cultures were grown statically at 30°C in lighted incubators for 3 days. Surface-lying mycelial mats were moved aside with sterile pipette tips, and broth was extracted for use in pre-screening for cellulase expression by dot blot analysis. Tissue culture plates were stored at 4°C until positive expressing transformants were identified. Following identification of positive expressing clones, the mycelial mats were transferred using sterile tweezers to the edge of PDH plates. These plates were incubated for 3 days at 30°C in lighted incubators to generate a lawn of spores. These spores were then struck out for single colonies on PDHX plates to ensure clonal populations. These colonies were again screened for Cel7A production, and positive expressing clones were again allowed to generate spore lawns on PD plates, and spore stocks were made using 20% glycerol. Stocks were frozen at −80°C.

### Cel7A purification

Fermentation broths (approximately 8 to 10 L) were harvested and sequentially vacuum-filtered through the following series: (1) Miracloth (EMD Biosciences, St. Charles, MO, USA), (2) approximately 2-μm glass fiber filter, (3) 1.1-μm glass fiber, and (4) a 0.45-μM PES membrane. This filtered broth was then concentrated by tangential ultrafiltration with a 10,000-Da MWCO. The broths were roughly concentrated from 8.0 L to 150 to 200 mL. The final concentrated volume was exchanged with at least 1.0 L of 20 mM Bis-Tris pH 6.5 to remove residual peptides and other low molecular weight debris. This concentrate was then re-filtered to 0.2 μM. This filtrate was adjusted to 1.5 M (NH_4_)_2_SO_4_ for hydrophobic interaction chromatography (HIC) and vacuum filtered through 0.2-μm PES, then loaded onto a 26/10 Phenyl Sepharose Fast Flow column. Buffer (A) was 20 mM Bis-Tris pH 6.5 and buffer (B) was 20 mM Bis-Tris pH 6.5, 2 M (NH_4_)_2_SO_4_. After washing out the unbound sample at 80% B, elution was via a descending buffer B gradient from 80% (1.6 M) to 0% over eight column volumes. Active fractions were identified by a *p*NP-lactose (*p*NPL) activity assay (*p*NPL at 2 mM in 50 mM acetate pH 5.0) where 100 μL of *p*NPL added to each well of a 96-well plate, followed by 25.0 μL of each fraction. The plate was then incubated 30 min at 45°C. Reactions were quenched with 25 μL 1.0 M NaCO_3_ and the absorbance at 405 nm (*A*_405_) was measured. Standard curve concentrations range from 0 to 250 μM *p*NP.

*p*NPL-active fractions were pooled and concentrated and then desalted and exchanged into 20 mM Bis-Tris pH 6.5 buffer using two sequential Superdex 25 Hi-Prep desalting columns. The desalted protein was loaded onto a Source 15Q 10/100 Tricorn anion exchange column and run at 0% to 50% B over 30 column volumes. Buffers were 20 mM Bis-Tris pH 6.5 (A) and the same buffer plus 1.0 M NaCl (B). *p*NP-L activity was followed again to identify active fractions. SDS-PAGE and αCel7A immunoblotting (described elsewhere) was performed to assess purity. The final stage of purification consisted of size exclusion chromatography (SEC) using a 26/60 Superdex 75 column and a 20 mM acetate pH 5.0 buffer with 100 mM NaCl in the mobile phase.

### Differential scanning calorimetry

Thermal stability was evaluated by DSC using a Microcal model VP-DSC calorimeter (Microcal, Inc., Northampton, MA, USA). Data analysis was completed by Origin for DSC software (Microcal). Samples were prepared containing 50 μg/mL protein in a 20 mM acetate pH 5.0 buffer with 100 mM NaCl. Calorimeter scan rate was 60°C/h over a range of 30°C to 110°C.

### Cel7A enzyme activity assay

Cellobiohydrolase activity is measured as the conversion of the cellulose fraction of a sample of a standard dilute acid-pretreated corn stover by the cellobiohydrolase used in conjunction with two other enzymes at standard loadings: (1) the endoglucanase *Acidothermus cellulolyticus* E1 (Cel5A, catalytic domain, Y245G mutant) loaded at 1.894 mg/g of biomass cellulose and (2) the chromatographically-purified *beta*-glucosidase from *Aspergillus niger*, loaded at 2.0 mg/g biomass cellulose.

The standard biomass substrate used in the activity assays is NREL dilute acid-pretreated corn stover P050921, washed first with water and then with 20 mM acetic acid/sodium acetate buffer, pH 5.0, until the pH of the (buffer) decantate is within 0.03 units of pH 5.00. From a slurry of this washed biomass material (approximately 9.0 mg biomass/mL of pH 5.0, 20 mM acetate buffer containing 0.02% sodium azide to retard microbial growth), a series of biomass substrate aliquots are prepared in 2.0-mL high-performance liquid chromatography (HPLC) vials, in such a way that each vial contains 8.5 mg biomass cellulose (which, given that the ‘glucan’ content of this batch of pretreated stover is 59.1%, requires 14.38 mg of biomass per digestion vial). Biomass dry weights for each batch of assay vials were verified by dry weight determinations on a group of five samples co-pipetted into pre-tared vials. The acceptable relative standard deviation for a batch of biomass assay aliquots is 1% or less, with a preferred value of 0.8% or less. Adjustment of these biomass assay aliquots to a 1.7-mL final volume results in a cellulose concentration of 5.0 mg/mL.

Cellobiohydrolase assays were carried out in triplicate vials at 40°C, pH 5.0 in 20 mM azide-containing acetate buffer, with continuous mixing by inversion at 10 rpm while immersed in a water bath. At various times during the digestion, vials are removed from the rotator and representative 100-μL samples containing both solids and liquid are removed from the well-stirred contents and diluted 18-fold into glass HPLC vials. The primary digestion vials are immediately resealed and returned to the rotator in the assay 40°C water bath so that the assay digestions may continue. The vials containing the withdrawn and diluted samples of digestion mixture are then crimp-sealed and immersed in a boiling water bath for 10 min to denature the enzymes and terminate the reaction. The contents of the boiled time sample vials are then syringe-filtered (0.2-micron Acrodisc) into a third set of vials for sugar analysis by HPLC on a BioRad HPX-87H column operated at 65°C with 0.01 N H_2_SO_4_ as eluent at 0.6 mL/min and refractive index detection. Values for individual sugar concentrations in the digestion vials are back-calculated from the values measured by HPLC and then used to construct saccharification progress curves in terms of percent of conversion of biomass cellulose.

## Abbreviations

AEC: anion exchange chromatography
CBH1: cellobiohydrolase I
CML: complete media with lactose
DSC: differential scanning microcalorimetry
E1: endoglucanase I (*Acidothermus cellulolyticus*)
EG1: endogluconase I (*Hypocrea jecorina*)
HIC: hydrophobic interaction chromatography
HPLC: high-performance liquid chromatography
MA: Mandels Andreotti minimal medium
MAG: Mandels Andreotti minimal medium with 5% glucose
MAL: Mandels Andreotti minimal medium with 5% lactose
MOPS: 3-(n-morpholino)propanesulfonic acid
NHEJ: non-homologous end joining
PD: potato dextrose
PDH: potato dextrose with hygromycin
PDHX: potato dextrose with Hygromycin and Triton X-100
*p*NP: p-Nitrophenol
*p*NP-L: 4-nitrophenyl β-D-lactopyranoside
PVDF: polyvinylidene difluoride
SEC: size exclusion chromatography
